# Resilience of hop (*Humulus lupulus* L.) to salinity, heat and drought stresses: A mini-review

**DOI:** 10.3389/fpls.2022.1064922

**Published:** 2022-11-30

**Authors:** Roberto Marceddu, Alessandra Carrubba, Mauro Sarno

**Affiliations:** Department of Agricultural, Food and Forest Sciences, University of Palermo, Palermo, Italy

**Keywords:** abiotic stress, multiple stresses, crop performances, plant physiology, proteomics

## Abstract

Over recent years, the cultivation of hops (*Humulus lupulus* L.) has spread widely in the Mediterranean, also affecting the southern regions of Spain and Italy with a typical semi-arid climate. Several and recent studies have investigated the responses of this species to the main abiotic stresses, which is an aspect of absolute relevance to the knowledge of the adaptive capacity of hops to the growing conditions of a new cultivation environment. Moreover, given the fact that hops’ phytochemical composition is determined primarily by genetic and environmental factors, and that the species is perennial, the lack of knowledge on the effects of abiotic stress could be reflected in subsequent years, which means multi-year economic risks. This review work therefore aims to showcase, based on an in-depth investigation of the available literature, the response of hop to the main abiotic stresses, and the effect of these on productive and qualitative crop performances. The data presented will be useful to the understanding of constraints and to the identification of useful coping strategies to the cultivation of hops in semi-arid Mediterranean environments.

## Introduction

According to the prediction on climate models for the southern Mediterranean regions, a decrease in winter precipitation and an increase in the number of heat waves compared to previous decades will take place in next years (Beniston et al., 2007). Indeed, Earth surface temperature is expected to rise gradually on a global scale, and major changes are likely to occur in the hydrological and energy cycles (IPCC, 2001). Therefore, in the next decades, mankind will probably experience dramatic and threatening changes in regional extreme weather and climate events. Being “fixed” to their growth substrate, plants are supposed to be particularly exposed to changes in the frequency and intensity of extreme events (i.e., heat waves, heavy precipitation, droughts, *etc.*) (Christensen and Christensen, 2003; Goyette et al., 2003; Beniston, 2004; Schär et al., 2004; Ulbrich et al., 2001). An additional constraint is linked to the frequent simultaneous occurrence of two, or three, abiotic (and biotic) stressors, whose combination is mostly lethal to crops (Mittler, 2006), as much as that this “combined stress” is considered as a new and special complex stressor (Bai et al., 2018).

However, almost all vascular plants have developed in time several morphological and physiological adaptations to enable their survival under harsh conditions. Open field crops are almost always subjected to stressors coming from the surrounding environment, and that is the reason why it’s very hard that field crops on farms achieve their full yield potential, and huge yield gaps are highlighted, e.g., when field crops are compared to crops grown for experimental purposes (Lobell et al., 2009; Van Ittersum et al., 2013).

On the other side, the need to assess the climatic effects on quality is particularly relevant for some crops in which technological quality derives mainly from the aromatic-sensory and gustatory components (Ahmed et al., 2014).

**Figure 1:**
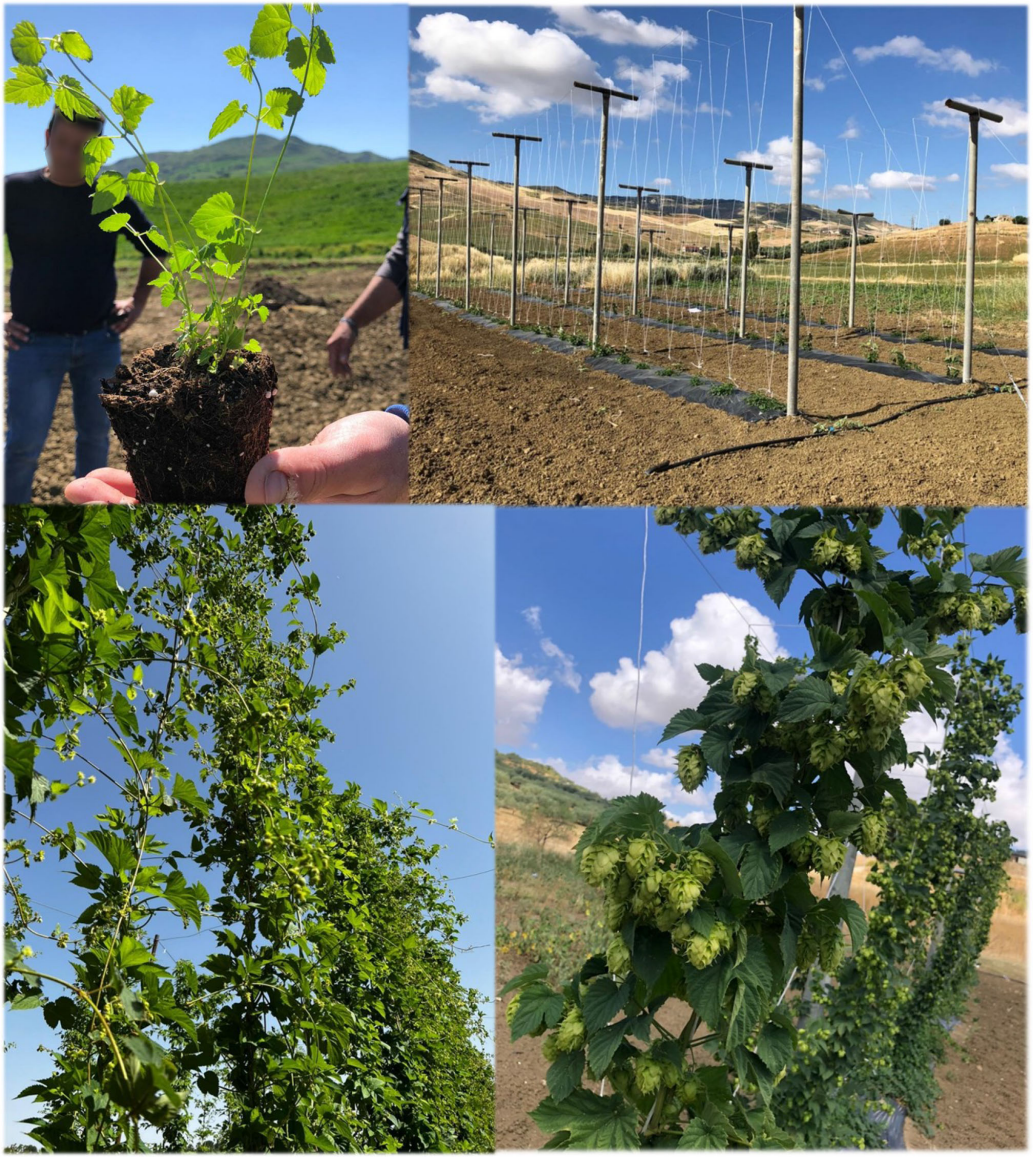
Pictures taken during the first year of hop cultivation in a Sicilian semi-arid environment at the “Sparacia” farm (37°38’07” N; 13°45’47” E; 450 m a.s.l.), Department of Agricultural, Food and Forest Sciences (D/SAAF), University of Palermo.

**Figure 2:**
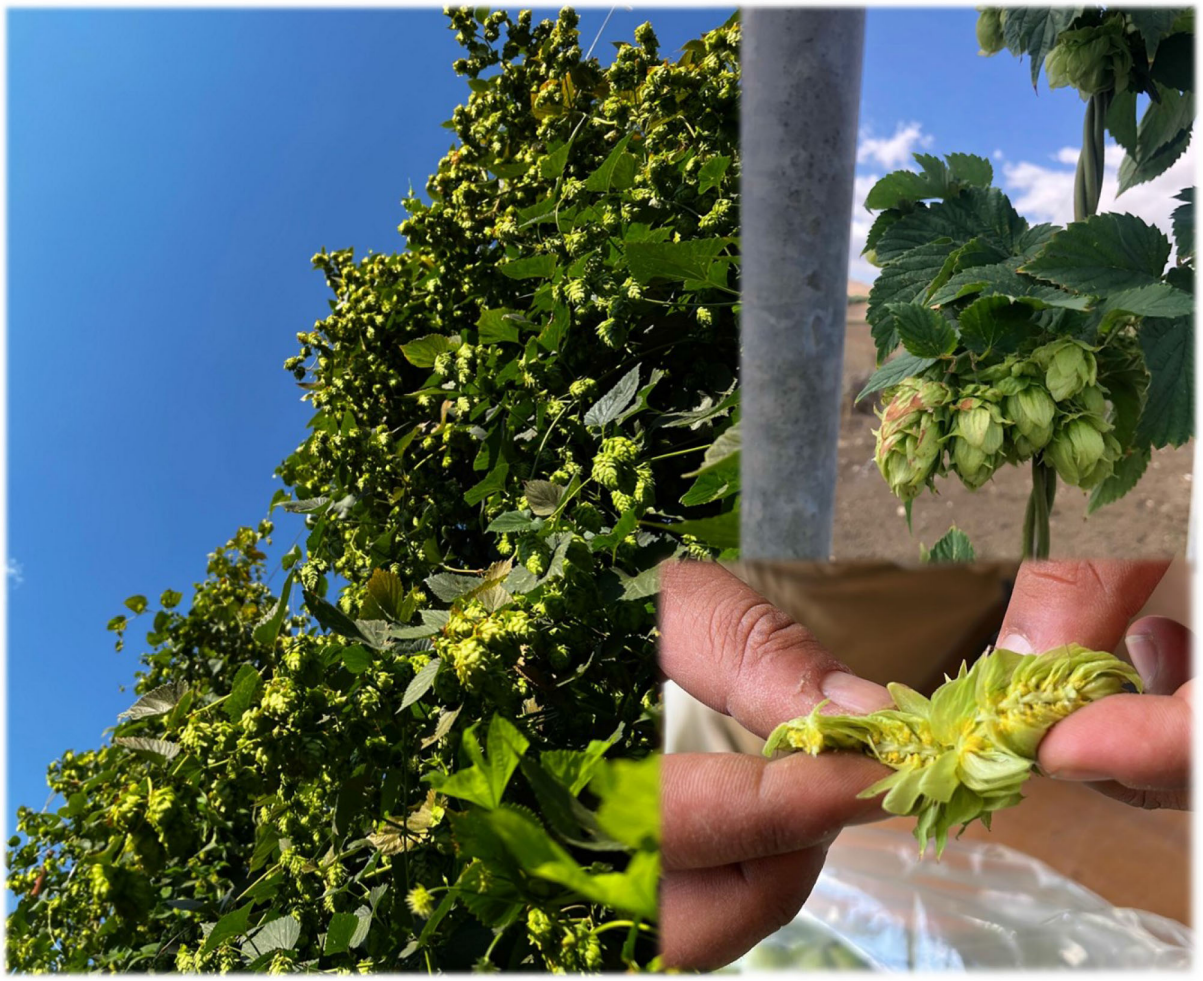
Hop bines before 2022 harvest at the “Sparacia” farm (37°38’07” N; 13°45’47” E; 450 m a.s.l.), Department of Agricultural, Food and Forest Sciences (D/SAAF), University of Palermo.

In hop (*Humulus lupulus* L.) several breeding programs have focused on improving agronomic performances, introducing characteristics such as resistance to biotic and abiotic stressors, reduced growth capacity (“dwarf” varieties), together with the improvement of yields and organoleptic characteristics of production (Hampton et al., 2001). To date, although numerous studies have already confirmed the excellent productive and qualitative response of hops grown in the Mediterranean environments (Mongelli et al., 2015; Rossini et al., 2016; Ruggeri et al., 2018; Marceddu et al., 2020; Rossini et al., 2021; **Figures 1**, **2**), there’s still the lack of knowledge on the effects of abiotic stresses on hop yield and quality. Since hop is a perennial crop, abiotic stresses might reflect in cone yields in subsequent growing seasons, representing a multi-year economic risk for farmers who decide to invest in this crop in a new environment such as the southern regions of the Mediterranean.

Hops are mainly cultivated for the beer industry as a source of secondary metabolites (i.e., α-acids, β-acids and aromatic oils), which give the characteristic bitterness and aroma, as well as antimicrobial properties to the brewing products (Neve, 1991). These substances are contained in the lupulin glands that develop mainly in the female inflorescences, and their amount increases as reproductive plant stages go on (i.e., from the appearance of cones to their full growth) (de Keukeleire et al., 2007).

Several studies have been carried out on the factors influencing the biosynthesis of these compounds. The biosynthesis of α-acids, β-acids and prenylated flavonoids involves extremely complex biosynthetic pathways (Goese et al., 1999; Paniego et al., 1999; Okada and Ito, 2001), that can be affected in many steps by several external factors, such as area of cultivation, phytosanitary status and age of crops (Jelínek et al., 2012; Matoušek et al., 2016; Morcol et al., 2020; Eriksen et al., 2021). It appears that, as with other plant secondary metabolites, both the genotype and the environment play a key role in determining their final concentration (Almaguer et al., 2014; Moore et al., 2014; Morcol et al., 2020).

This review work, therefore, aims to present the response of hop to three common abiotic stresses (salinity, heat, and drought), and the effects of these on crop productive and qualitative performances.

## Salinity stress

Among all the abiotic stressors, salinity is one of the major threats to crops’ productivity in semi-arid environments, due to its increased occurrence in farms with irrigated crops (Flowers, 2004). Salt stress persistence throughout plants’ growth cycles might lead to co-occurrence with other stressors, either abiotic or biotic, way more than drought or heat stress, which often occurs at various intermittent periods either preceding or following pathogen infection (Kissoudis et al., 2014). In general, all plants respond to saline stress with morphological and physiological modifications, responsible for the adaptation of the osmotic potential, thanks to the involvement of different genes and pathways (Julkowska and Testerink, 2015). Also, plant hormones appeared to have a key role in the response to salinity stress as reported by several researchers in different plant species (Golldack et al., 2014; Ryu and Cho, 2015; Tao et al., 2015).

However, limited research has been devoted to studying the basis of salinity tolerance in hops. Unquestionably, this species might adapt to multiple hostile conditions, determined by the presence of abiotic stressors such as salinity, activating all those mechanisms already well defined for most vascular plants, i.e., the alteration of cytoplasmic free Ca^2+^, the activation of Ca^2+^/calmodulin-dependent kinase, the production of secondary signaling molecules such as reactive oxygen species (ROS) and abscisic acid (ABA), and the activation of the salt overly sensitive (SOS) pathway for regulation and maintenance of ion homeostasis (Julkowska and Testerink, 2015).

In a field study with hops cultivars over a range of different cultivation sites, Dabbous-Wach et al. (2021) assessed the good acclimatization of German hop cultivars to the Corsican environment one year after planting, even though high levels of soil salinity were detected in most of the growing sites. A noticeable change in plant morphology was detected after the first year of cultivation: some plants showed a shortening of side shoots, and, considering the lower number of cones in the side shoots per plant, also reduced cone yields. From the phytochemical point of view, the essential oil of these genotypes showed a higher amount of myrcene (characterized by spicy and balsamic herbal notes) and α−humulene (woody notes), and lower α−selinene (herbal notes). Hence, a definite compositional and aroma difference was assessed between hops grown in the coastal areas and others from inner Corsica; although salinity could not be claimed as the only cause of these variations, it was certainly involved in the combined co-presence of the high salinity of soils, water scarcity, and wind, acting as a multiple stressor on cultivated plants. Interestingly, it appeared that not all the varieties reacted in the same way, as they have not been directly improved for salinity resistance, showing also very important information for growers to make a proper choice of hop cultivars that suit better to the local growing conditions, and that would be consistent with the outcomes of a recent research carried out in a semi-arid Mediterranean environment (Carrubba et al., 2022).

## Drought and heat stress

Numerous molecular and metabolic-related studies found that plants’ responses to the combination of heat and drought stress are unique and should be evaluated jointly rather than individually (Pnueli et al., 2002; Rizhsky et al., 2002; Rizhsky et al., 2004; Suzuki et al., 2005; Potopová et al., 2021). That is the reason for these stressors to be presented jointly in this paragraph.

In a Mediterranean environment, characterized by high temperatures throughout all hop growth season, an inverse correlation was shown between plant growth and productivity and heat accumulation, especially in the vegetative development stages (Marceddu et al., 2020). High temperatures (HT) and low-water (LW) stress during the growing season have consistently been shown to decrease hop cone yield and bitter acid content of cones (Srečec et al., 2004; Mozny et al., 2009; Nakawuka et al., 2017; Donner et al., 2020).

A recent study carried out by Donner et al. (2020) found a significant negative correlation between summer air temperature and α−acids content in several Czech cultivars (cv. “Saaz”, “Sladek” and “Premiant”). Mozny et al. (2009) also highlighted a decrease in cone yield in growing seasons with low precipitation and a decreased α-acids content in cv. “Saaz” hops during high-temperature years. Similar reductions in cones and α-acids yields under combined LW and HT conditions were also found in the cv. “Aurora” in Croatia (Srečec et al., 2004) and Slovenia (MacKinnon et al., 2020). In a study carried out by Nakawuka et al. (2017) in the Washington State (U.S.), a significant decrease in cone yields under reduced irrigation was assessed, even though no significant effect was assessed on bitter acid content in several American varieties (e.g., “Mt Hood”, “Columbus”, “Chinook”, and “Willamette”).

Nevertheless, limited research has been carried out on hop mechanisms and structural traits resulting from drought stress (Gloser et al., 2013; Korovetska et al., 2014; Korovetska et al., 2016).

On a general basis, it is well known that the main effects of drought stress on plant growth and development are determined by water relation disorders as well as modification of water use efficiency, with a major impact on the relative water content in green tissues, leaf water potential, osmotic potential, pressure potential and transpiration rate (Farooq et al., 2009; Farooq et al., 2012). Changes in the pH, ABA, and sulphate concentration in xylem sap were suggested as long-distance drought signals also for hop plants (Gloser et al., 2013; Korovetska et al., 2014; Korovetska et al., 2016), even though there is still no certainty about the role of these metabolites in the drought response of the species. In a trial carried out by Kolenc et al. (2016), the drought stress response of two Slovenian hop cultivars grown in pots was assessed by combining physiological studies and proteomic analysis. According to these findings, hop plants showed decreased transpiration rate and water potential during reduced water availability, experiencing a decrease in photosynthesis due to stomatal and non-stomatal limitation and a strong decrease in photosynthetic proteins and proteins of the energetic metabolism, affecting plant fitness in general. Also, a very interesting study was carried out by Eriksen et al. (2021) who looked at physiological traits and differential gene expression in leaf, stem, and root tissue in plants of the cv “Cascade” exposed to HT stress, LW stress, and a combination of both. In the above experiment, the cultivation trial took place in growth chambers where the imposed stress conditions were able to impress substantial changes to the transcriptome. Significant reductions in the expression of numerous genes were detected, which resulted in a decrease in agronomically important secondary metabolite biosynthesis, e.g., bitter acids. However, as reported by the same Authors, other studies found no reductions in α−acids content under LW stress (Nakawuka et al., 2017) or cultivar-specific reactions to LW and HT stresses (Donner et al., 2020), suggesting possible cultivar-based differences in the temperature tolerance range, that could be exploited to develop breeding lines with increased resilience to abiotic stress.

Heat stress-wise, it is known that plants exposed to high temperatures might manifest various symptoms, deriving from the drastic limitation of their photosynthetic activity (Berry and Bjorkman, 1980). Allakhverdiev and coworkers (2008) identified three components of the photosynthetic system that are sensitive to heat damage, namely the photosystems themselves, the ATP-generating electron transport chain, and the carbon assimilation process. According to other research also, heat resulted to affect photosystem II by causing the dissociation of Manganese (Mn) from the oxygen-evolving complex, but also by disrupting the distribution of absorbed light energy from the light-harvesting complex (Enami et al., 1994; Enami et al., 1998; Nash et al., 1985; Pastenes and Horton, 1996).

The disruption of membrane fluidity caused by heat, also, determines the breakdown of the thylakoid membrane integrity, which leads to disruptions in the electron transport chain and ATP synthesis (Gounaris et al., 1983; Inaba and Crandall, 1988). Moreover, HT stress was found responsible for the destruction of the Rubisco activase protein, leading to the inactivation of the carboxylating enzyme and, therefore, to the disruption of carbon assimilation (Salvucci et al., 2001; Salvucci and Crafts-Brandner, 2004; Sharkey, 2005). In six hop cultivars exposed to HT (within a range of temperatures from 15 to 45°C), Eriksen et al. (2020) highlighted that all the tested plants achieved maximal carbon assimilation at temperatures ranging from 21 to 39°C without the availability of water being a limiting factor. When the temperatures reached and overpassed 41°C, all plants experienced severe stress, showing decline in carbon assimilation, due to multiple effects on the cell, including damage to photosystem II (PSII), damage to membrane integrity as reflected in electrolyte leakage at high temperatures, and declines in Rubisco activity probably due to deactivation of Rubisco-activase enzyme. According to these findings, “Cascade” and “Southern Brewer” appeared to be better candidates for use as breeding lines to improve abiotic stress tolerance than “Chinook”, which appeared to be particularly susceptible to extreme heat stress.

## Conclusions

Biosynthesis pathways of secondary metabolites in hops, and therefore concentration levels in cones, proved very sensitive to stress conditions such as salinity, LW and HT. Such sensitivity is variable according to many factors, including genotype, intensity and duration of stress, the simultaneous occurrence of two or more stressors, but also phenology of the plant. For example, the stage of cone development is one of the most crucial for flavor compounds biosynthesis in hops, therefore representing a key moment for the success of hop cultivation. Even though HT stress is difficult to avoid in the field, LW stress should be minimized in irrigated systems during this period. In this sense, agrotechnical care is an essential tool to obtain satisfactory production levels, above all when those to be managed are new crops in new areas of cultivation. The findings from the research above might represent valuable information for growers developing hopyards in the increasingly warm regions of the Mediterranean. However, it also emerged that some hop genotypes are better adapted than others to environmental constraints, and are, therefore, more suitable to highly demanding cultivation areas. The proper choice of the right variety given the environmental context is a key point to successfully manage this challenging crop.

## Author contributions

AC conceived the project. RM and AC wrote the manuscript. RM, AC, and MS revised the manuscript. All authors contributed to the article and approved the submitted version.

## Conflict of interest

The authors declare that the research was conducted in the absence of any commercial or financial relationships that could be construed as a potential conflict of interest.

## Publisher’s note

All claims expressed in this article are solely those of the authors and do not necessarily represent those of their affiliated organizations, or those of the publisher, the editors and the reviewers. Any product that may be evaluated in this article, or claim that may be made by its manufacturer, is not guaranteed or endorsed by the publisher.
